# Diagnostic Accuracy of Fine-Needle Aspiration Cytology in the Diagnosis of Parotid Gland Swellings

**DOI:** 10.7759/cureus.86177

**Published:** 2025-06-16

**Authors:** Nandakumar B. M., Sandeep S, Madhukumar H. V., Srikantaiah C Hiremath, Padma Priya

**Affiliations:** 1 General Surgery, Ramaiah Medical College, Bengaluru, IND; 2 General Surgery, Indira Gandhi Medical College and Hospital, Shimla, IND; 3 Pathology, Ramaiah Medical College, Bengaluru, IND

**Keywords:** diagnostic test accuracy, fine needle aspiration cytology (fnac), fnac of parotid lesions, parotid swelling, pleomorphic adenomas

## Abstract

Background

Parotid gland swellings are the most common salivary gland lesions. Fine-Needle Aspiration Cytology (FNAC), apart from being the first-line diagnostic tool in the evaluation of parotid gland swellings, plays an integral role in planning both surgical and non-surgical therapy.

Objectives

To determine the diagnostic accuracy of FNAC in the evaluation of parotid gland swellings.

Methodology

Medical records of all patients who underwent surgery for parotid gland lesions at Ramaiah Medical College and Hospital, Bengaluru, Karnataka, since 2010 were retrospectively analyzed. Demographic data, FNAC reports, and final histopathology reports were extracted, assessed, and statistically analyzed.

Results

Out of the 193 patients who underwent surgery for parotid gland swelling during the study period, 112 cases were included in the study. The cohort consisted of 67 male and 45 female patients, with a mean age of 50 years. FNAC diagnosed 94 patients with benign disease and 18 patients with malignant disease. Final histopathological analysis of post-surgical specimens revealed that 86 cases had benign pathology, while 26 had malignant disease. Pleomorphic adenoma was the most common pathology observed in both FNAC and histopathological analysis.

The diagnostic performance of FNAC in identifying parotid gland pathology was as follows: sensitivity, 65.38%; specificity, 98.83%; accuracy, 91.07%; positive predictive value (PPV), 94.4%; negative predictive value (NPV), 90.42%; positive likelihood ratio (LR+), 55.88; and negative likelihood ratio (LR-), 0.35.

Conclusions

Our findings demonstrate that FNAC has good accuracy in distinguishing benign lesions from malignant ones. FNAC is a readily available, repeatable, inexpensive, minimally invasive, and relatively painless outpatient procedure with no known serious complications. While a relatively low sensitivity may limit its standalone diagnostic value, its high specificity, PPV, NPV, and LR+ underscore its importance as a valuable diagnostic tool in the evaluation of parotid gland swellings.

## Introduction

Tumors of the salivary gland account for up to 3-4% of all head and neck tumors. About 80% of salivary gland tumors occur in the parotid gland, and 75% of them are benign [1]. The typical tumor of the salivary gland is one in which the benign variant is less benign than usual benign tumors, and the malignant variant is less malignant than typical malignant tumors [2]. The parotid glands are a pair of major salivary glands. The diagnostic methods for parotid gland swellings mainly consist of ultrasound and fine-needle aspiration cytology (FNAC). Given the wide diversity of tumors arising in the salivary glands, many diagnostic challenges exist, including the lack of a uniform reporting system for salivary gland cytopathology. These challenges are further amplified by the fact that several low-grade carcinomas demonstrate significant morphologic overlap with their benign counterparts [3]. This is compounded by the fact that most parotid FNAs are not ultrasound-guided. Until 2017, a dichotomous method of reporting parotid FNAC was in use, categorizing parotid masses as either benign or malignant. In 2017, the Milan System for Reporting Salivary Gland Cytopathology was proposed as a more stratified method. Although revised in 2023, universal acceptance of this system is still lagging. Given the heterogeneity and overlapping histopathological features of salivary gland tumors, some studies have evaluated the role of core needle biopsy as an efficient diagnostic modality. In our institutional retrospective study, we aim to gain insight into the utility of FNAC for parotid swellings and explore the need for additional diagnostic modalities.

## Materials and methods

Objective of the study

To determine the diagnostic accuracy of FNAC in the evaluation of parotid gland swellings.

Study design

This retrospective study included all patients who underwent parotidectomy from January 2014 to October 2024, after meeting the inclusion and exclusion criteria.

The study was conducted at Ramaiah Medical College and Hospitals after receiving ethical approval from the Ethics Review Board, Ramaiah Medical College and Hospitals (ERB registration number: EC/NEW/INST/2023/KA/0244), with approval number MSRMC/EC/SP-03/11-2024.

To achieve statistically significant results that could be compared with previous studies (absolute precision of 6% and a 95% CI), a minimum of 105 subjects was required.

Inclusion and exclusion criteria

Patients of both sexes aged 18 years and above who had undergone parotidectomy were included in the study as per protocol. Patients who had not undergone a preoperative FNAC of the parotid swelling, and FNACs reported as inconclusive, were excluded.

Methodology

The case files of all patients who underwent parotidectomy at Ramaiah Medical College and Hospitals during the proposed study period were extracted from the centralized Medical Records Department. All relevant data required for the study (demographic data, FNAC reports, histopathology reports) were extracted as per protocol and entered into the proforma. The data was then statistically analyzed.

Statistical analysis

Demographic data were analyzed and summarized in terms of mean. Descriptive statistics for malignancy and benign histopathology were reported in percentages. Sensitivity, specificity, positive predictive value (PPV), and negative predictive value (NPV) were calculated to determine the accuracy of FNAC.

## Results

A total of 193 patients underwent parotidectomy during the study period. Eighty-one patients were excluded from the study, as FNAC was either not performed or reported as inconclusive. One hundred and twelve patients were included in the study.

**Figure 1:**
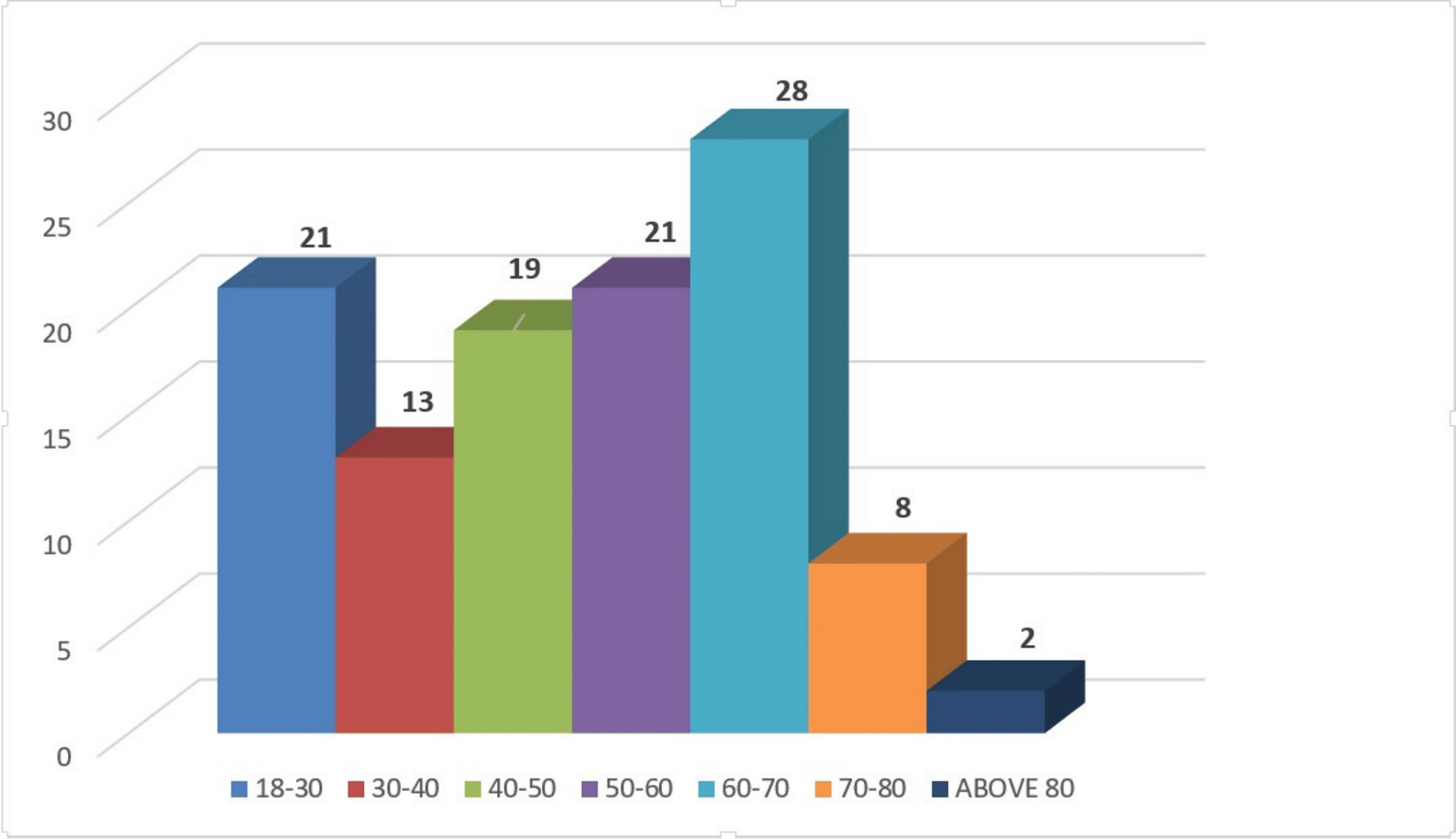
Distribution of patients’ ages in the study.

Patients’ ages ranged from 18 to 86 years, with the majority in the 5th and 6th decades of life, and a mean age of 50 years. There were 67 (59.82%) male and 45 (40.17%) female patients, with a male-to-female ratio of approximately 3:2. The distribution of patient age is depicted in Figure 1.

Ninety-four (83.92%) of the 112 parotid FNAs were reported as benign, while 18 (16.07%) were reported as malignant. The distribution of various FNAC findings for parotid swellings is shown in Table 1. Pleomorphic adenoma was the most common benign entity on FNAC, followed by Warthin’s tumor. Among FNACs reported as positive for malignancy, mucoepidermoid carcinoma was the most common malignant cytology.

**Table 1:** Distribution of benign and malignant fine-needle aspiration cytology (FNAC) diagnoses.

Diagnosis	Number of Cases (%)
Benign Cytology	
Pleomorphic adenoma	58 (61.70%)
Warthin’s tumor	16 (17.02%)
Adenomatous lesion	1 (1.06%)
Benign cystic lesion	11 (11.70%)
Inflammatory lesion	5 (5.31%)
Lipoma	1 (1.06%)
Monomorphic adenoma	2 (2.12%)
Total	94 (100%)
Malignant Cytology	
Acinic cell carcinoma	1 (5.56%)
Adenoid cystic carcinoma	1 (5.56%)
Mucoepidermoid carcinoma	6 (33.33%)
Positive for malignancy	10 (55.56%)
Total	18 (100%)

On final histopathology, 26 (23.21%) specimens were found to harbor malignancy, while the remaining 86 (76.78%) were benign. The distribution of final histopathology findings of parotid swellings is shown in Table 2. Similar to FNAC reporting, pleomorphic adenoma, followed by Warthin’s tumor, were the most common benign pathologies on histological analysis. Mucoepidermoid carcinoma was the most common malignant entity on histopathology.

**Table 2:** Distribution of benign and malignant histopathological diagnoses.

Diagnosis	Number of Cases (%)
Benign Histopathology	
Pleomorphic adenoma	52 (60.46%)
Warthin’s tumor	20 (23.25%)
Oncocytoma	3 (3.48%)
Basal cell adenoma	1 (1.16%)
Chronic sialadenitis	3 (3.48%)
Cystadenoma	1 (1.16%)
Kimura’s disease	1 (1.16%)
Lymphangioma	1 (1.16%)
Lymphoepithelial cyst	1 (1.16%)
Monomorphic adenoma	1 (1.16%)
Sialolipoma	1 (1.16%)
Myoepithelioma	1 (1.16%)
Total	86 (100%)
Malignant Histopathology	
Acinic cell carcinoma	3 (11.53%)
Adenoid cystic carcinoma	1 (3.84%)
Adenosquamous carcinoma	1 (3.84%)
Non-Hodgkin’s lymphoma	1 (3.84%)
Mucoepidermoid carcinoma	18 (69.23%)
Myoepithelial carcinoma	1 (3.84%)
Necrotic carcinoma	1 (3.84%)
Total	26 (100%)

Nine (9.57%) of the 94 (83.92%) FNACs reported as benign turned out to be malignant, while one (5.55%) of the 18 (16.07%) FNACs reported as malignant turned out to be benign. These discordant FNAC and histopathology cases are described in Table 3. Mucoepidermoid carcinoma was the most common malignant pathology misinterpreted as a benign entity on FNAC.

**Table 3:** Discordance between fine-needle aspiration cytology (FNAC) and histopathology. FNAC: Fine-needle aspiration cytology.

Age	Sex	FNAC Diagnosis	Histopathological Diagnosis
74	F	Pleomorphic adenoma	Myoepithelial carcinoma
53	M	Pleomorphic adenoma	Mucoepidermoid carcinoma
32	F	Inflammatory lesion	Non-Hodgkin’s lymphoma
24	F	Monomorphic adenoma	Mucoepidermoid carcinoma
67	F	Monomorphic adenoma	Adenoid cystic carcinoma
43	M	Pleomorphic adenoma	Mucoepidermoid carcinoma
57	M	Benign cystic lesion	Mucoepidermoid carcinoma
51	M	Warthin’s tumor	Mucoepidermoid carcinoma
35	M	Benign cystic lesion	Mucoepidermoid carcinoma
19	F	Adenoid cystic carcinoma	Pleomorphic adenoma

The correlation between FNAC and final histopathology, assessed in the 112 patients included in the study, is shown in Table 4 and was used for statistical analysis.

**Table 4:** Contingency table between cytological and histopathological diagnoses. FNAC: Fine-needle aspiration cytology.

	Malignant Histology	Benign Histology	Total
FNAC - Malignant	17	1	18
FNAC - Benign	9	85	94
Total	26	86	112

FNAC demonstrated a sensitivity of 65.38% and a specificity of 98.83%. The overall accuracy of FNAC in diagnosing malignancy was 91.07%. The PPV was 94.40%, and the NPV was 90.42%. The likelihood ratio of a positive test result was 55.88, while the likelihood ratio of a negative test result was 0.35, where “positive” indicated malignancy. These results are tabulated in Table 5.

**Table 5:** Metrics of diagnostic evaluation of fine-needle aspiration cytology (FNAC) in parotid swellings.

Metric	Abbreviation	Value
Sensitivity	Sn	65.38%
Specificity	Sp	98.83%
Accuracy	Acc	91.07%
Positive predictive value	PPV	94.40%
Negative predictive value	NPV	90.42%
Positive likelihood ratio	LR⁺	55.88
Negative likelihood ratio	LR⁻	0.35

## Discussion

Parotid swellings are a common diagnosis in the surgical outpatient department. Due to their relative infrequency, the diagnosis and treatment of parotid swellings pose a challenge to surgeons. The majority of parotid swellings are neoplastic, of which 75% are benign. The usual symptom is a slow-growing swelling, often noticed by a friend or family member, without any other associated symptoms. History and clinical examination usually cannot distinguish between malignant and benign lesions. Although rare, features suggesting malignancy include involvement of the facial nerve, rapid growth, and skin infiltration.

The need for imaging a parotid mass is not universally accepted among surgeons, as many consider FNAC to be sufficient for planning surgical intervention. Ultrasound is a good first-line investigation for salivary masses. It provides excellent discrimination between normal parotid tissue and the neoplastic component, which typically appears hypoechoic compared to the surrounding parotid tissue [4]. A common drawback is the difficulty in visualizing the deep lobe of the gland. An added advantage is that ultrasound can guide FNAC or FNAB. The utility of CT and MRI is limited and is indicated only when clinical presentation raises concern for malignancy or when FNAC yields malignant cytology. FNAC has been widely accepted as the most important diagnostic test for the management of parotid lesions. Despite its widespread use, concerns about false-negative results and variable sensitivity and specificity have raised questions about its reliability over the decades. The primary limitation lies not with the technical aspects of FNAC but with the vast heterogeneity of parotid lesions.

One of the major stumbling blocks is that most salivary gland tumors arise from or differentiate toward the same cell lines: epithelial (ductal and/or acinar) and abluminal (myoepithelial and/or basal cell). This results in considerable overlap at all diagnostic levels, compounded by the fact that each of these cells can undergo various metaplastic changes (e.g., oncocytic, squamous, clear cell, sebaceous, chondroid) [5].

Consistent with existing literature, our study found that parotid swellings were more common in males than females, with a male-to-female ratio of 3:2. The majority of patients were in the 5th and 6th decades of life.

FNAC was the standard preoperative investigation in all patients. Pleomorphic adenoma was the most common benign pathology, and mucoepidermoid carcinoma was the most common malignancy. Benign and malignant lesions constituted 86 (76.78%) and 26 (23.21%) cases, respectively. Our findings are concordant with existing literature, which states that up to 75% of parotid swellings are benign.

Over the decades, many researchers have studied the utility of FNAC in the preoperative diagnosis of parotid swellings. The value of FNAC has been questioned due to its low sensitivity and high rate of false-negative results. While its diagnostic accuracy ranges from 78% to 98%, sensitivity is generally lower and more variable (52%-98%) than specificity (56%-100%) [6]. This may be due to variations in study settings. Referral centers often have dedicated cytopathologists who specialize in salivary gland lesions, leading to better recognition of subtle pathological differences and improved diagnostic accuracy. Recent studies evaluating the diagnostic utility of FNAC in parotid swellings are summarized in Table 6.

**Table 6:** Recent studies evaluating the diagnostic utility of fine-needle aspiration cytology (FNAC) in parotid swellings.

Study (Year and Author)	Sensitivity (%)	Specificity (%)	Accuracy (%)
2023, Mourouzis C et al. [6]	98.3	87.5	97.1
2022, Gundamaraju DV et al. [7]	62.5	94.1	84
2022, Rameeza A and Hemalata M [8]	95	85	–
2022, Hajiioannou J et al. [9]	98	93	–
2021, Yildiz S et al. [10]	59.09	97.85	93.75
2020, Dhanani R et al. [11]	88.9	97.9	95.8

In Table 6, we observe an increase in the diagnostic specificity of salivary gland lesions. This suggests that the false-positive rates of FNAC have decreased, although sensitivity still remains low in some studies.

In our study, FNAC had a sensitivity of 65.38% and a specificity of 98.83%. The accuracy of FNAC in diagnosing malignancy was 91.07%. The PPV for malignancy was 94.40%, and the NPV was 90.42%. The likelihood ratio of a positive test result was 55.88, and the likelihood ratio of a negative test result was 0.35, where "positive" indicated malignancy.

Our study showed a low sensitivity of FNAC in diagnosing malignancy, consistent with findings from previous studies. A 9% discordance rate was noted between FNAC and histopathology. Among these, mucoepidermoid carcinoma was the most frequently misdiagnosed malignancy on FNAC. Mucoepidermoid carcinoma is composed of varying proportions of mucous, intermediate, and epidermoid cells, and a variable amount of cystic degeneration, which can mimic benign pathology. According to Cohen MB et al., mucoepidermoid carcinoma is one of the most difficult and challenging lesions to diagnose cytologically [12].

Among the 9 patients with malignant histology, 5 underwent revision total parotidectomy, 6 received radiation therapy, and 1 patient with lymphoma underwent chemotherapy.

Our study underscores the fact that FNAC alone cannot reliably distinguish between malignant and benign lesions preoperatively. Considerable histological overlap, along with the dependency of FNAC on the skills and experience of both the operator and cytopathologist, the preparation and fixation of slides, and the adequacy of sample material, all contribute to the difficulty in making a definitive preoperative diagnosis [6]. The lack of a standardized classification system was aptly addressed by the proposal of the Milan System for Reporting Salivary Gland Cytopathology (MSRSGC).

The MSRSGC standardizes the cytological evaluation and reporting of salivary gland lesions and uses a six-tiered classification system rather than a dichotomous “yes/no” approach [3]. Each category in the MSRSGC is associated with an implied risk of malignancy (Table 7).

**Table 7:** Diagnostic categories of the MSRSGC and their associated risk of malignancy (ROM). MSRSGC: Milan System for Reporting Salivary Gland Cytopathology; ROM: Risk of malignancy; SUMP: Salivary gland neoplasm of uncertain malignant potential.

Diagnostic Category	Risk of Malignancy (ROM)
1) Nondiagnostic	25%
2) Non-neoplastic	10%
3) Atypia of undetermined significance	20%
4a) Neoplasm - Benign	<5%
4b) Neoplasm - SUMP	35%
5) Suspicious for malignancy	60%
6) Malignant	90%

Although proposed in 2017 and revised in 2023, universal acceptance of the MSRSGC among cytopathologists is still limited [13].

Compared to FNAC, core needle biopsy yields better tissue for analysis and has demonstrated higher sensitivity (96%) and specificity (100%), with only 1.6% of specimens being considered non-diagnostic. The main concerns with core biopsy are the risks of facial nerve injury, tumor seeding, and fistula formation [4]. However, these risks can be mitigated by using USG guidance.

In 2015, Eom HJ et al. studied the value of USG-guided core needle biopsy in differentiating benign from malignant salivary gland lesions. They concluded that ultrasound-guided core needle biopsy had 100% sensitivity, specificity, and accuracy in this context. No patients developed hematoma, infection, facial nerve injury, or tumor seeding [14]. In 2022, Kazemi MA et al. conducted a study comparing FNA and core needle biopsy under ultrasonographic guidance for detecting malignancy and for tissue-specific diagnosis of salivary gland tumors [15]. They reported that ultrasound-guided core needle biopsy is superior to USG-guided FNA in detecting and characterizing malignant salivary gland tumors, and it could emerge as the diagnostic method of choice for patients presenting with a salivary gland mass. No complications were reported in their study. The utility of core needle biopsy, however, needs to be further validated through more prospective trials before it can be considered a standard preoperative investigation.

## Conclusions

Our findings demonstrate that FNAC has good accuracy in distinguishing benign lesions from malignant ones. It is a readily available, repeatable, inexpensive, minimally invasive, and relatively painless outpatient procedure with no known serious complications. While its relatively low sensitivity may limit its standalone diagnostic value, its high specificity, PPV, NPV, and positive likelihood ratio (LR⁺) underscore its importance as a valuable diagnostic tool in the evaluation of parotid gland swellings.
